# BBGRE: brain and body genetic resource exchange

**DOI:** 10.1093/database/bat067

**Published:** 2013-09-27

**Authors:** Joo Wook Ahn, Abhishek Dixit, Caroline Johnston, Caroline M. Ogilvie, David A. Collier, Sarah Curran, Richard J. B. Dobson

**Affiliations:** ^1^Department of Cytogenetics, Guy's and St Thomas NHS Foundation Trust, London, SE1 9RT, UK, ^2^MRC Social, Genetic and Developmental Psychiatry Centre, Institute of Psychiatry, King's College London, De Crespigny Park, London, SE5 8AF and ^3^NIHR Biomedical Research Centre for Mental Health at South London and Maudsley NHS Foundation, London, SE5 8AF

## Abstract

Studies of copy number variation (genomic imbalance) are providing insight into both complex and Mendelian genetic disorders. Array comparative genomic hybridization (array CGH), a tool for detecting copy number variants at a resolution previously unattainable in clinical diagnostics, is increasingly used as a first-line test at clinical genetics laboratories. Many copy number variants are of unknown significance; correlation and comparison with other patients will therefore be essential for interpretation. We present a resource for clinicians and researchers to identify specific copy number variants and associated phenotypes in patients from a single catchment area, tested using array CGH at the SE Thames Regional Genetics Centre, London. User-friendly searching is available, with links to external resources, providing a powerful tool for the elucidation of gene function. We hope to promote research by facilitating interactions between researchers and patients. The BBGRE (Brain and Body Genetic Resource Exchange) resource can be accessed at the following website: http://bbgre.org

**Database URL:** http://bbgre.org

## Background and significance

Clinical genetic laboratories are increasingly using high-resolution microarray-based genomic analyses, usually array comparative genome hybridization (aCGH), as first-line diagnostic tests to detect copy number variants (CNVs) in individuals referred for congenital abnormalities and developmental disabilities (1). As well as improving genomic diagnosis overall, these high-resolution analyses have enabled discovery of novel CNVs associated with genetic syndromes and complex disorders such as autism, epilepsy and intellectual disability (2, 3). Despite the many successes so far, there is a need to better exploit the volume of clinical and genomic data that is being accumulated in diagnostic genetic laboratories (typically thousands of patients tested per year per centre) and to make these data available to the wider scientific and clinical genetic community.

A number of resources are already available to the community: CHOP CNV holds CNV data derived from a study of ∼2000 healthy individuals (4), and DGV (5) holds an aggregated set of control data from ∼12 000 healthy individuals. DECIPHER (6) and ISCA (7) databases each hold aggregated data from ∼5000 and ∼28 000 phenotypically abnormal individuals, respectively.

## Objective

The Brain and Body Genetic Resource Exchange (BBGRE) was set up to provide CNV data and clinical phenotype of patients referred for genetic testing at a single regional genetics laboratory. This resource is of particular interest to local researchers, as those patients who have given informed consent are easily accessible; the majority of individuals are from a single catchment area and re-contact of patients is included on the consent form. The resource may also be of particular interest to researchers wishing to concentrate on this geographically defined population. This facet of the data may be a disadvantage for the same reasons depending on the hypothesis in question; however, this data set can also be integrated into meta-analyses and aggregate studies.

Specifically, this article describes a database created to house this clinical and genetic data, as well as a web-based interface for interrogation and interpretation of BBGRE data. Furthermore, the website allows registered users to submit research project proposals, which are forwarded to the steering committee for approval.

## Materials and methods

### CNV detection and interpretation

We have previously described the implementation of aCGH as a test for genome imbalance (8); in short, CNVs are detected using an Agilent (USA) 60K platform with a median resolution of 120 kb (AMADID 028469), using a patient versus patient hybridization strategy. Full details of protocols, analysis and interpretation are presented in Ahn *et al.* (8). Briefly, samples were co-hybridized with other samples mismatched for phenotype and matched for sex. Analysis was performed using Agilent algorithm ADM-2, threshold 6 and a 3-probe minimum aberration call; a further analysis using ADM-1 was carried out to maximize detection of mosaicism (9). Imbalances of regions represented in DGV by at least three non–BAC-based studies were classified benign. The clinical significance of remaining imbalances was assessed by examining the functional content of the region of imbalance, referencing known benign and pathogenic CNVs (ISCA, DECPIHER, OMIM) and an internal clinical database of previously tested individuals (Moka). All samples with imbalances that were of potential clinical significance were re-tested using G-banded karyotyping, QF-PCR, FISH, custom MLPA (10) or a repeat array.

### Data collection

The referral process for clinical aCGH at Guy’s Hospital requires clinicians to fill out a genetic testing referral form, and patients provide informed consent on a signed BBGRE consent form. The consent form allows patients to specify whether they agree to be contacted for research studies and/or to allow their DNA sample to be used for research. The referral form includes a checklist of referral reasons, comprising a comprehensive list of neurodevelopmental disorders, as well as a range of other medical and congenital conditions (http://bbgre.org/info/accessing-data). The checklist is the preferred method for indicating the clinical phenotype, although free text entry is also allowed. On receipt of samples at the laboratory, details from the referral and consent form are recorded in a bespoke LIMS (Moka) behind the Guy’s & St Thomas’ NHS Trust firewall.

Once aCGH testing is complete, the phenotype, consent and CNV data are collected for patients that are found to carry potentially clinically significant CNVs. A unique BBGRE ID is generated for each patient before the data are anonymized. A key table linking the BBGRE ID to patients is stored within Moka. The anonymized data are routinely transferred via sFTP from Moka to the BBGRE server as flat files, mirroring the data structure of the BBGRE database tables. A simple script on the BBGRE server is scheduled to import any data when received.

### Database design and population

**Figure 1.**
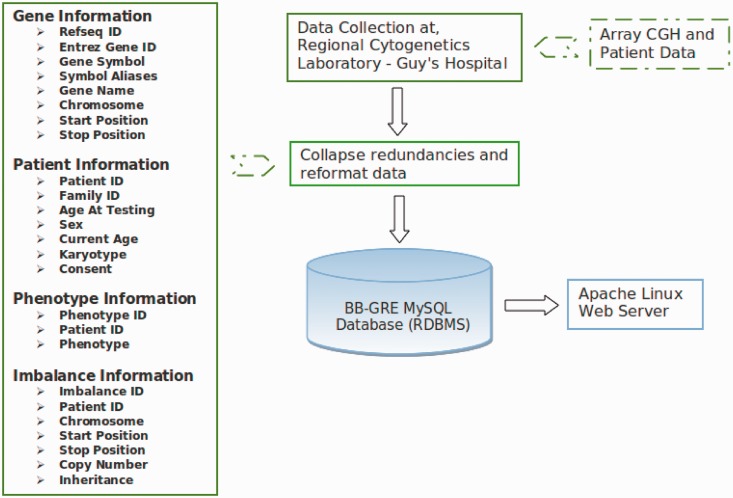
Outline of BBGRE infrastructure.

An overview of the BBGRE infrastructure and data flow is shown in Figure 1. The resource comprises a Linux server with a MySQL database backend. To populate the BBGRE database, each table within Moka is exported and transferred as tab-separated flat text files. A Perl script parses the flat files to remove trailing spaces, escape special characters and convert chromosome identifiers into integers (X- >23, Y- >24). The script then imports the files into the MySQL database. Each table in the BBGRE database is deleted and then repopulated with each new Moka version. Gene annotation is taken from HGNC and NCBI RefSeq (currently hg 19). Web pages are served up to the users’ browser via an Apache web server using Perl/CGI and jQuery/Ajax.

### Data interrogation

Users access BBGRE via the BBGRE website, http://bbgre.org. Here, users are able to interrogate the database for CNV and basic demographic data. The search interface allows use of combinations of criteria including genomic location, gene content, CNV inheritance patterns and patient age and gender. A search with CNV specific criteria returns a table of individual CNVs, whereas a search with demographic criteria returns a table of patients. These tables can be sorted via column headers, and iterative searching is also available. To aid further analysis, tables can be exported as tab separated file (.tsv) and searches can be saved as web browser bookmarks. Each table also provides links to patient details pages where all the demographic and CNV information for individual patients is displayed. To help with interpretation of these data, links are provided to view CNVs in the UCSC Genome Browser (11). A UCSC session is used to provide a predefined set of tracks and viewing configuration that best provides a genomic context for the CNV in question. A custom track showing all CNVs contained in BBGRE is also displayed to show context within BBGRE.

Users are also able to apply for ‘advanced user access’, which enables searching of clinical phenotype data and also allows submission of research project proposals. One key aspect of this resource is the potential availability of research participants within a relatively small geographical area, which, for example, would simplify the logistics associated with further deep phenotyping of cases. As the steering committee acts as the gatekeeper between researchers and patients, this registration is necessary. All applications are considered by the steering committee via a polling system integrated into the BBGRE website. Successful application results in advanced user status, which enables the user to access clinical phenotype data. These phenotype data can then be searched as per the other criteria described earlier in text. Advanced users are able to track the status of submitted projects as they progress through the approval process.

### Discussion and conclusion

The current version of the BBGRE database (April 2013) contains 4092 cases considered to carry CNVs of potential clinical significance, and 4908 imbalances (2429 with inheritance information) derived from a total pool of >10 000 individuals referred for testing at a single regional genetics laboratory at Guys Hospital, London. The database will be updated every 6 months as it is expected to grow by 1000 cases per year. Analytical methods for CNV detection and interpretation of imbalances are, therefore, far more consistent than the aggregated data sets available elsewhere, which can be based on different platforms and interpretation tools. BBGRE, therefore, provides a high-quality data set for clinical interrogation and for the basis of research studies. Data contained within BBGRE are regularly updated as more patients are tested clinically.

Microdeletions in the NRXN1 gene have been associated with a range of neurodevelopmental disorders, including autism spectrum disorders, schizophrenia, intellectual disability, speech and language delay, epilepsy and hypotonia. Using the BBGRE tool, we found exonic deletions in the NRXN1 gene, predominantly affecting the alpha isoform, in patients with a range of neurodevelopmental disorders referred for diagnostic cytogenetic analysis (12). Patients have a range of phenotypes including developmental delay, learning difficulties, attention-deficit hyperactivity disorder, autism, speech delay, social communication difficulties, epilepsy, behaviour problems and microcephaly.

Other users of the BBGRE database have also been able to identify patients who have contributed to research studies. A patient carrying an intragenic deletion of NRXN3 present in this collection was one of the four index cases of such deletions in autism spectrum disorder cases (13). It has also facilitated identification of a male sex bias in 46 patients carrying 16p13.11 CNVs (14).

By way of example, we describe the search to identify patients with NRXN3 imbalances described earlier in text. A user would browse to the main web page http://bbgre.org and navigate to the search page using the links on the left navigator menu. The gene identifier NRXN3 should then be entered into the ‘GENE’ text box. As the user types the characters into the text box, suggestions for genes present in the database will be offered through a ‘predictive text’ mechanism. The user then clicks the ‘search’ button, and seven patients will be returned in the results (data release 2). Inspecting the GenomicSize column shows us that six patients look to be chromosome 14 trisomy mosaic patients due to the size of the imbalance. Clicking on the ‘details’ button for patient BBGREID:113108 opens up a new browser window that gives more patient details including gender, age, age at testing and details of this and any other imbalances this patient has. Clicking on the ‘view’ button displays this patient’s imbalance on the UCSC genome browser alongside other patients from BBGRE as well as annotation that includes OMIM, DECIPHER and DGV. Having identified the patient(s) of interest, the user can then apply for access to the phenotypes by registering as an advanced user. Application for advanced user status involves clicking on the left navigation menu and completing a short web form that includes PI contact details, institution and a short project summary (≤200 words). As well as providing access to the phenotypes, the advanced user registration is the route through which a user would begin the process of accessing the patient for recruitment into further studies.

A process is underway to label the clinical phenotypes within BBGRE according to medical subject headings terms (National Library of Medicine). This will shortly be incorporated into the web resource, enabling users to perform phenotype searches that exploit the hierarchical nature of the medical subject headings definitions.

In summary, the BBGRE resource provides easy access to and user-friendly search of CNV genotype and clinical phenotype data from patients referred for genetic testing. Initial data mining can be performed on the website and the results exported and/or viewed on the UCSC Genome Browser for further analysis.

This resource will prove useful for clinicians and researchers, and contributes to the understanding of genotype/phenotype correlations and the elucidation of gene function. Patients are from a single catchment area, and we hope to promote research by facilitating interactions between researchers and patients.

## Funding

A strategic partnership of the National Institute for Health Research (NIHR) specialist Mental Health Biomedical Research Centre (BRC) at the South London and Maudsley NHS Foundation Trust and King’s College London, and the comprehensive BRC at Guy’s and St Thomas NHS Foundation Trust and King’s College London. Funding for open access charge: National Institute for Health Research (NIHR) Mental Health Biomedical Research Centre (BRC) at the South London and Maudsley NHS Foundation Trust and King’s College London.

*Conflict of interest*. None declared.
